# Air pollution accelerates the development of obesity and Alzheimer’s disease: the role of leptin and inflammation - a mini-review

**DOI:** 10.3389/fimmu.2024.1401800

**Published:** 2024-06-12

**Authors:** Clara Machado Campolim, Bianca Camilo Schimenes, Mariana Matera Veras, Young-Bum Kim, Patricia Oliveira Prada

**Affiliations:** ^1^ Department of Internal Medicine, School of Medical Science, State University of Campinas (UNICAMP), Campinas, SP, Brazil; ^2^ Division of Endocrinology, Diabetes, and Metabolism, Department of Medicine, Beth Israel Deaconess Medical Center, and Harvard Medical School, Boston, MA, United States; ^3^ School of Applied Sciences, State University of Campinas (UNICAMP), Limeira, SP, Brazil; ^4^ Laboratory of Environmental and Experimental Pathology LIM05, Department of Pathology, School of Medicine, University of São Paulo (USP), São Paulo, SP, Brazil; ^5^ Obesity and Comorbidities Research Center, Campinas, SP, Brazil; ^6^ Department of Structural and Functional Biology, Institute of Biology (IB), University of Campinas, Campinas, SP, Brazil

**Keywords:** air pollution, particulate matter 2.5, leptin, neuroinflammation, TLR4, obesity, Alzheimer’s disease

## Abstract

Air pollution is an urgent concern linked to numerous health problems in low- and middle-income countries, where 92% of air pollution-related deaths occur. Particulate matter 2.5 (PM_2.5_) is the most harmful component of air pollutants, increasing inflammation and changing gut microbiota, favoring obesity, type 2 diabetes, and Alzheimer’s Disease (AD). PM_2.5_ contains lipopolysaccharides (LPS), which can activate the Toll-like receptor 4 (TLR4) signaling pathway. This pathway can lead to the release of pro-inflammatory markers, including interleukins, and suppressor of cytokine signaling-3 (SOCS3), which inhibits leptin action, a hormone that keeps the energy homeostasis. Leptin plays a role in preventing amyloid plaque deposition and hyperphosphorylation of tau-protein (p-tau), mechanisms involved in the neurodegeneration in AD. Approximately 50 million people worldwide are affected by dementia, with a significant proportion living in low—and middle-income countries. This number is expected to triple by 2050. This mini-review focuses on the potential impact of PM_2.5_ exposure on the TLR4 signaling pathway, its contribution to leptin resistance, and dysbiosis that exacerbates the link between obesity and AD.

## 1 Introduction

Air pollution is one of the significant environmental risks associated with morbidity and premature death (1). This concern is even more urgent in low- and middle-income countries, where 92% of air pollution-related deaths occur (1, 2). One of the most harmful components of air pollutants is particulate matter, classified according to its aerodynamic diameter, whose size determines its distribution and potential health impacts.

The classes of particles based on their aerodynamic diameter typically include particulate matter 2.5 (PM_2.5_) - particles with a diameter of 2.5 micrometers or less (3) - which can remain suspended in the air for extended periods. Its toxicity relies on its concentration and composition. Their surface area-to-volume ratio makes them more effective at absorbing other toxic substances in the air. Considering the toxicity of PM_2.5_, The World Health Organization’s Air Quality Concentration Guideline established in 2005 that the mean concentrations of PM_2.5_ should not surpass 10 µg/m^3^ per year. However, data from 2019 indicated that 90% of the population consistently faced concentrations overcoming the guidelines, resulting in fatalities and illnesses. Thus, in 2021, the guideline set a new goal of 5 µg/m^3^ annually (2).

In urban areas, the primary source of PM_2.5_ is vehicular emissions, composed of sulfates, nitrates, ammonia, carbons, hydrogen, lipopolysaccharides (LPS), metals, and water (4, 5). PM_2.5_ primarily enters the body through inhalation via the upper airways. It reaches the lungs, and the immune system recognizes it, causing local inflammation to spread to the systemic circulation and tissues (6–9). In addition, PM_2.5_ might directly access the central nervous system (CNS) via the nasal epithelium and the olfactory bulb, inducing neuroinflammation (10, 11). The inflammation caused by PM_2.5_ exposure could be the link between air pollution exposure (PM_2.5_) and obesity, which is a disorder that, in turn, increases an individual’s risk of developing dementia.

This mini-review will focus on the implications of PM_2.5_ exposure, particularly its potential to activate the immune system in the brain areas that regulate feeding, cognition, memory, and behavior. This activation could contribute to the pathology of obesity and Alzheimer’s disease (AD). Furthermore, we will explore the possible involvement of leptin in this mechanism.

## 2 PM_2.5_ infiltration into the central nervous system causing inflammation

PM_2.5_ exposure caused tissue-like damage and pro-inflammatory responses in the hippocampus, cortex, and hypothalamus (12–16), potential areas involved in the pathophysiology of obesity and dementia. *In vivo* and *in vitro* studies have demonstrated that long- and short-term exposure to PM_2.5_ increases inflammatory markers such as tumor necrosis factor-alpha (TNF-α), interleukin 1 beta (IL-1β), interleukin 6 (IL-6), Toll-like receptor 4 (TLR4), and nuclear factor–kappa B (NFkB) in different brain areas (12, 17, 18).

Since PM_2.5_ may contain LPS (19, 20), a component of gram-negative bacteria membranes, and a primary agonist of TLR4, our immune cells could recognize it as a pathogen after it crosses the blood-brain barrier (BBB). TLR4 is a receptor from the innate immune system. Its activation recruited myeloid differentiation factor 88 (Myd88). This association leads to the inhibitor kappa B kinase (ikkB) phosphorylation, releasing NFkB to the cell nucleus to start the production and secretion of pro-inflammatory proteins (21, 22).

Studies have shown that six months of PM_2.5_ exposure increased the expression of pro-inflammatory genes and activated the Ikk/NF-κB pathway in the arcuate nucleus of the hypothalamus (ARC) of control mice (13), an area related to feeding regulation. Evidence demonstrated that PM_2.5_ exposure led to microglia activation by upregulating ionized calcium-binding adaptor molecule 1 (Iba-1) expression in the hypothalamus, hippocampus, and cortex (23–25). One of our recent studies revealed that maternal exposure to PM_2.5_ induces hypothalamic inflammation in offspring (16), suggesting that in addition to the direct exposure damage seen in other studies, prenatal exposure to PM_2.5_ also induces inflammation (15, 16, 18, 24). Diesel exhaust (DE) is also a significant component of PM_2.5_, and its acute exposure increased interleukin one alpha (IL-1α), IL-1β, interleukin 3 (IL-3), IL-6, and TNF-α levels in the olfactory bulb and hippocampus, in addition to activate microglia in the hippocampus of control mice (26).

Besides obesity (24) and AD (27), PM_2.5_ led to other conditions involving neuroinflammation, including Ischemic Stroke (28), Parkinson’s disease (29), and Type 2 Diabetes *Mellitus* (T2DM) (30, 31). Therefore, PM_2.5_ might trigger systemic inflammation and elevated levels of pro-inflammatory cytokines in the CNS, thereby being a risk factor for metabolic diseases and neurodegenerative disorders.

## 3 The effects of air pollution in the development of Alzheimer’s disease

AD is a progressive neurodegenerative disease that primarily affects memory, cognition, and behavior and gradually worsens over time. While specific medications can alleviate some symptoms, there is currently no cure for AD (32). The accumulation of amyloid beta (Aβ) plaques and hyperphosphorylated tau-protein (p-tau) in the brain are characteristics of AD (33, 34). In 2018, Alzheimer’s Disease International estimated that approximately 50 million people worldwide were affected by dementia, with a significant proportion living in low— and middle-income countries. This number is expected to triple by 2050 (35).

The primary risk factors for developing AD are typically over 65 and carrying at least one apolipoprotein E (APOE) ϵ4 allele. In the preclinical phase, alterations in neurons, microglia, and astroglia contribute to the progression of the disease before cognitive impairments become noticeable. Neuroinflammation, vascular changes, aging, and non-efficient astroglial cell waste clearance play crucial roles in this cellular disease landscape (35).

The presence of Aβ aggregates in the brain initiates a response from microglia, which releases harmful elements like nitric oxide (NO), reactive oxygen species (ROS), pro-inflammatory cytokines, and chemokines, causing neuron damage. These microglia triggered by Aβ are often co-localized with amyloid plaques in AD. In this state, microglia may contribute to the hyperphosphorylation of p-tau (36, 37).

The activation of microglia TLR4 by Aβ aggregates facilitates its clearance and function as a natural protected event. However, accumulating Aβ aggregates due to self-sustaining cycles generating Aβ diminishes TLR4 capacity, overwhelming the body’s natural mechanisms for resolving Aβ aggregates (38, 39). Therefore, the protective effect of TLR4 is present in the initial stages of aggregation, but prolonged TLR4 activation may contribute to Aβ deposition (40).

Stewart et al. (41), proposed that Aβ interacts with the TLR4 through a CD36-TLR4-TLR6 complex. This mechanism involves the recognition of Aβ by cluster of differentiation 36 (CD36), which then initiates signaling through TLR4 and toll-like receptor 6 (TLR6), activating downstream inflammatory pathways. The CD36-TLR4-TLR6 complex increases the IL-1β expression, a cytokine prominently found in AD plaques (41).

IL-1β is a maker of neuroinflammation and could be triggered by PM_2.5_ (42). As discussed, constant exposure to PM_2.5_, which contains LPS, might cause prolonged TLR4 activation (19, 20), ultimately contributing to Aβ deposition. Evidence suggests that PM_2.5_ exposure could be a risk factor for AD (10, 25, 43).

In a study by Lee et al. (10), AD-model mice exposed to PM_2.5_ for 3 months (11.38 μg/m^3^ mean concentration) showed an increase in p-tau levels at olfactory bulb, as well as MDA, an oxidative stress biomarker, in the hippocampus and olfactory bulb. These effects indicate the meaningful pathway through which PM_2.5_ enters and propagates oxidative damage. The olfactory bulb could be the starting point for the damage caused by PM_2.5_, and the lack of damage to other brain regions or biomarkers could be due to low-level exposure (10). A study with an AD mouse model (APP/PS1) found that exposure to PM_2.5_ at 25.8 μg/m^3^ for 3 months significantly increased Aβ plaque density in the hippocampus. This result suggests that chronic exposure to PM_2.5_ could worsen AD pathology (25).

Furthermore, Ning et al. (44) found that 1 month of exposure to PM_2.5_ (3 mg/kg) led to impaired learning and memory in young mice (4 weeks) after performing the Morris water maze test (44). Another study revealed that exposure to PM_2.5_ (3 mg/kg) for one month was enough to raise tau hyper-phosphorylation levels in the cortex of middle-aged mice. Remarkably, these elevated levels returned to baseline after the cessation of PM_2.5_ exposure (43). Collectively, these observations suggest that chronic PM_2.5_ exposure could be a crucial AD risk factor.

## 4 PM_2.5_-induced leptin resistance: implications for Alzheimer’s disease and obesity

Leptin is an anorexigenic hormone secreted by adipocytes in proportion to their mass that regulates satiety and energy expenditure through a molecular signaling cascade. This cascade involves binding to its receptor and subsequently activates the phosphorylation of signal transducer and activator of transcription 3 (STAT3). STAT3 translocates to the nucleus cell and stimulates the transcription of Pomc neurons while reducing Agrp/Npy secretion from neurons on the ARC, increasing satiety and energy expenditure (45–47). Besides the neurons of the hypothalamus, leptin receptor long isoform (LepRb) is present in the hippocampus, cortex, cerebellum, brainstem, and thalamus (48, 49). In these areas, leptin is involved in motivation, reproduction, growth, learning, and memory (50–52).

**Figure 1:**
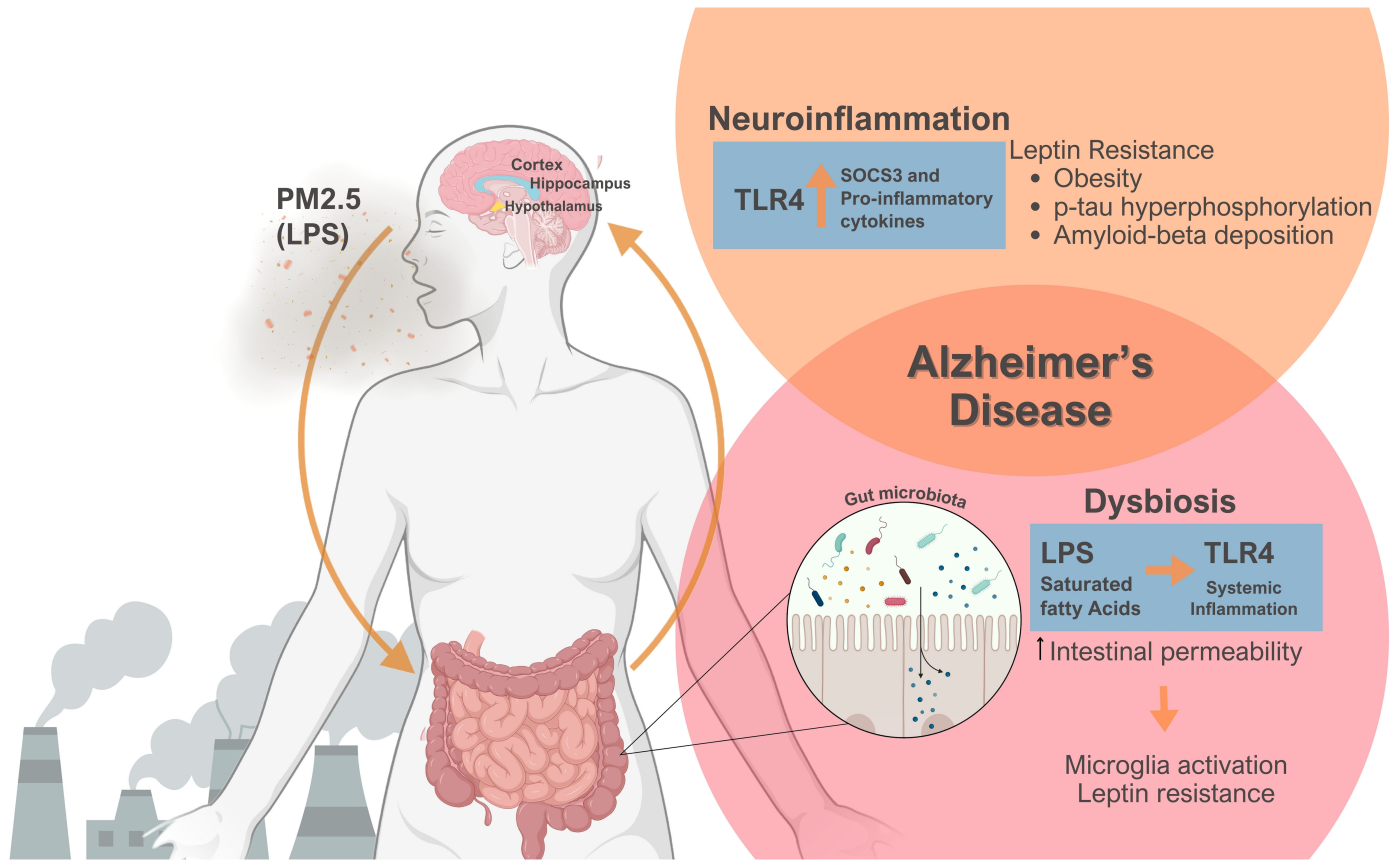
Emphasizes the role of PM_2.5_ exposure on the gut-brain axis, culminating in leptin resistance, obesity, and Alzheimer’s disease. PM_2.5_-derived Lipopolysaccharide (LPS) can engage Toll-like receptor 4 (TLR4) signaling, thereby triggering neuroinflammation via the production of pro- inflammatory cytokines and suppressor of cytokine signaling-3 (SOCS3), a physiological inhibitor of signal transducer and activator of transcription 3 (STAT3) phosphorylation leading to leptin resistance. Obesity and the lack of leptin action heightened tau-protein phosphorylation and amyloid-beta deposition. Simultaneously, PM_2.5_ exposure causes dysbiosis, changing the composition of gut microbiota. Combined with a poor diet, LPS, and Saturated Fatty Acids could provoke systemic inflammation, thereby increasing intestinal permeability. This scenario could activate microglia and worsen leptin resistance. Significantly, PM_2.5_ induces neuroinflammation and changes the gut microbiota, two phenomena crucial in Alzheimer’s disease pathogenesis. Figure created using BioRender.com.

A suppressor of cytokine signaling-3 (SOCS3) is a physiologic leptin signaling inhibitor typically expressed with leptin signaling. However, in the context of obesity and leptin resistance with the activation of the TLR4/Myd88/NFkB axis, an enhanced SOCS3 expression occurs, causing a pathologic leptin signaling inhibition (45, 53). PM_2.5_’s LPS content might activate the TLR4 signaling pathway in the various brain regions, including the hypothalamus, hippocampus, and cortex (40, 45, 54). This effect increases several pro-inflammatory cytokines and SOCS3 production, consequently causing leptin resistance (**Figure 1**) (21, 22, 45, 53). Therefore, unhealthy obesity is considered a state of low-grade systemic and hypothalamic inflammation, which could impair neuronal function (55, 56). Leptin resistance contributes to an obese state and promotes the formation of Aβ plaques and hyperphosphorylation of p-tau, which ultimately leads to the onset and progression of AD (**Figure 1**) (57–60).

Rodents with obesity displayed elevated circulating leptin concentrations. Despite higher leptin levels, the anorexigenic effect is lacking, suggesting leptin resistance (61, 62). Although age and genetics are well-established risk factors in AD pathogenesis, the link between leptin resistance, obesity, and the development of AD has been the subject of several studies (63–65).

Interestingly, individuals with AD had lower plasma leptin levels. Over 50% of subjects with mild cognitive impairment showed lower leptin levels than the control group (66). The APP/PS1 mouse model of AD had similar lower leptin levels (67). Together, these studies suggest that circulating leptin levels may play a role in cognitive impairment.

Not only are lower leptin levels associated with AD, but patients with AD exhibit increased leptin levels in the cerebrospinal fluid (CSF) and hippocampus while concurrently showing a decrease in its receptor, suggesting leptin resistance. Furthermore, a reduction in leptin receptor expression is evident even in cases where leptin levels are the same between groups (68, 69). The hippocampus is a complex brain structure that plays an essential role in learning, memory, cognition, and synaptic plasticity (70). It is AD’s first and primary affected area (69). In addition to the reduction of the leptin receptor expression in the hippocampus of AD rodent experimental models, such as old Tg2576, apoE4, and APP/PS1 mice, the STAT3 phosphorylation is also impaired in this area, as reviewed by McGregor et al. (48).

In control mice, leptin increases glutamate receptors long-term potentiation (LTP), improving excitatory synaptic transmission and decreasing long-term depression (LTD). This modification suggests that leptin influences the brain’s fundamental process underlying learning and memory. In APP/PS1 mice with lower leptin levels, supplementation with leptin improves the harmful effects of Aβ on hippocampal LTP and LTD, restoring normal synaptic function, increasing synaptic density, and ameliorating memory deficits (68).

*In vivo* and *in vitro* studies showed leptin injection attenuates Aβ toxic levels in hippocampus neuronal cells (57, 71). Other studies showed that activating the leptin signaling pathway in the AD mouse model decreases tau phosphorylation in the hippocampus, indicating that leptin has a possible protective effect (72, 73). Leptin protects against AD by reducing the expression of the β and γ-secretase enzymes. The β and γ-secretase enzymes made the amyloid precursor protein (APP) cleavage, assembling Aβ toxic structures. Therefore, they promote the initiation of amyloid plaque deposition and tau hyperphosphorylation, leading to neurodegeneration (57, 58). Together, the studies suggest that normal leptin signaling and action are beneficial to avoid AD progression. Human and mouse models of AD displayed lower leptin levels or leptin resistance, in which leptin has an incomplete effect.

In summary, elevated leptin levels contribute to reduced signaling response in obesity, leading to leptin resistance. In contrast, a lack of leptin alters essential brain functions currently under investigation for their potential impact on AD development, particularly obesity. In both obesity and AD scenarios, insufficient leptin action is a marked risk factor for neurodegenerative progression. Scientific research seeks to understand how leptin influences AD pathology and whether it holds promise as a therapeutic target.

## 5 The overlapping aspects of Alzheimer’s disease and obesity

AD and obesity share several environmental risk factors, such as a sedentary lifestyle, consuming a high-fat and high-sugar diet, and increased stress due to lack of sleep (74–76). Obesity in midlife is associated with cognitive reduction and memory and verbal and spatial ability impairments afterward (77–79). Exposure to lower education, poor nutrition, and family stressors during early years increases the risk of cognitive impairment and dementia in later life (80, 81).

Age and genetics are independent risk factors contributing to AD pathogenesis development (82). Individuals who develop obesity in midlife have a 33% higher incidence of AD (83). Neurodegenerative diseases often display obesity as a comorbidity in neurodegenerative diseases, as they share biomolecular mechanisms that can lead to brain damage. Both conditions show increased levels of pro-inflammatory cytokines, including IL-1β, IL-6, TNF-α, and leptin resistance. These conditions trigger neurodegeneration through neuroinflammation (12, 80, 84–86).

In addition to inflammation, obese individuals with elevated oxidative stress and mitochondrial dysfunction have an increased risk of developing AD (87, 88). Notably, elevated levels of APP in adipose tissue and of Aβ in the bloodstream have also been identified as significant contributors to this heightened (87, 89, 90).

In the review by Flores-Cordero et al. (33), they highlight that obesity resulting from high-fat diets (HFD) increases the risk of dementia, affecting cognitive abilities such as memory, attention, and executive functions, with neuroinflammation playing a crucial role. Studies on male Wistar rats fed with HFD displayed impaired memory. In contrast, assessments of rats fed with a high-fructose-high-coconut oil diet using the Morris water maze task revealed hippocampal-dependent learning and memory problems. These impairments were followed by molecular changes related to inflammation, stress, and central insulin resistance (33).

Similarly, a recent study by da Cruz Rodrigues et al. (91) revealed that obese mice aged 32 weeks lacking Low density lipoprotein receptor-related protein 1 (LRP1) in GABAergic neurons demonstrated impaired performance in learning and memory tasks, including the Water-T Maze and Spatial Novelty Y Maze compared to mice at 16 weeks of age. The aging mice exhibited diminished locomotor activity compared to their control counterparts. Also, these mice showed higher leptin levels and neurodegeneration indicators. These findings suggest that obesity may significantly influence cognitive function age-dependently (91).

In summary, the studies point to increased inflammation, Aβ and APP elevated levels, and leptin resistance as the sharing characteristics between obesity and AD. These elements contribute to neuroinflammation and neurodegeneration, which are associated with the development of AD.

## 6 PM_2.5_ exposure and gut microbiota dynamics: insights into obesity and Alzheimer’s disease

The gut microbiota is another aspect influencing obesity and AD pathology, potentially affected by environmental factors like PM_2.5_ and dietary choices.

A healthy gut microbiota shows significant diversity and variability in microorganisms (92, 93), a crucial regulator of energy balance. The alteration, known as dysbiosis, is associated with obesity, T2DM, colorectal cancer, and AD (94, 95).

Animal studies reveal that environmental pollutants notably disrupt the composition of the gut microbiota (96–98). Microbes ingested with PM_2.5_ induce inflammatory responses in local immune cells, increase intestinal permeability, and alter the gut environment, promoting the growth of specific microbial strains adapted to an inflammatory milieu (99).

Transplanting the gut microbiota from obese mice into germ-free animals increases fat mass, indicating that obese animals have a specific gut microbiota that promotes excess body weight accumulation (100, 101). Several diet factors might also change the gut microbiota, contributing to obesity induction. High-fat and high-sucrose diets are often deficient in essential vitamins and minerals and can induce adverse metabolic effects (102). A high-fat diet and a cafeteria diet led to leptin resistance and dysbiosis, which, in turn, altered the gut microbiota composition, increasing the intestinal permeability to LPS uptake. As a result, LPS and saturated fatty acids from the diet can activate TLR4, initiating systemic inflammation (**Figure 1**) (102, 103).

Studies on germ-free (GF) mice emphasize the role of gut microbiota in modulating immune responses and gene expression patterns associated with inflammation, including the microglia activation in the brain (95, 104, 105). Microglia from GF mice showed immature profiles and impaired immune responses compared to conventionally raised mice. In particular, transcription and survival factors usually suppressed in mature microglia were increased in GF mice. Recolonization of GF mice with fecal samples from AD patients activates microglia more significantly than samples from healthy donors (95, 106).

Evidence suggests that the development of AD may originate in the gut and then spread to the brain, reinforcing the idea of the gut-brain axis (107–110). Aβ1–42 oligomers injected into the gastric wall of mice migrate from the intestine to the brain. Additionally, bacterial strains such as *Escherichia coli* and *Salmonella enterica* produce extracellular amyloid fibers, which can initiate immune responses and enhance the formation of neuronal amyloid in the brain (95, 111, 112).

In summary, PM_2.5_ impacts obesity and AD by influencing the gut microbiota. Dysbiosis and nutrient-deficient diets lead to leptin resistance and exacerbated intestinal permeability (**Figure 1**), thereby triggering systemic inflammation. Additionally, the gut microbiota influences immune responses, including microglia function. Given the importance of the connection between the gut and AD, interventions targeting the gut microbiome are crucial in managing both obesity and AD.

## 7 Major conclusions

Our mini-review discusses the increasing prevalence of AD and obesity as global health challenges, exploring the impact of PM_2.5_ exposure on these illnesses. We examine evidence suggesting that immune system activation leads to leptin resistance, a critical facet in these conditions. Additionally, we discuss the need for further research on PM_2.5_-induced inflammation, neurodegeneration, and the protective role of leptin on AD pathogenesis. Finally, we highlight the urgent action to reduce air pollution and its associated health risks.

## Author contributions

CC: Conceptualization, Writing – original draft. BS: Writing – original draft. MV: Writing – review & editing. Y-BK: Writing – review & editing. PP: Writing – review & editing.
